# *PcDWF1*, a pear brassinosteroid biosynthetic gene homologous to *AtDWARF1*, affected the vegetative and reproductive growth of plants

**DOI:** 10.1186/s12870-020-2323-8

**Published:** 2020-03-06

**Authors:** Xiaodong Zheng, Yuxiong Xiao, Yike Tian, Shaolan Yang, Caihong Wang

**Affiliations:** 1 College of Horticulture, Qingdao Agricultural University, No. 700 Changcheng Road, Qingdao, 266109 China; 2 Qingdao Key Laboratory of Genetic Improvement and Breeding in Horticulture Plants, Qingdao, 266109 China

**Keywords:** Brassinosteroids, *PcDWF1*, Vegetative and reproductive growth, *Pyrus ussuriensis*, *Nicotiana tabacum*

## Abstract

**Background:**

The steroidal hormones brassinosteroids (BRs) play important roles in plant growth and development. The pathway and genes involved in BR biosynthesis have been identified primarily in model plants like *Arabidopsis*, but little is known about BR biosynthesis in woody fruits such as pear.

**Results:**

In this study, we found that applying exogenous brassinolide (BL) could significantly increase the stem growth and rooting ability of *Pyrus ussuriensis*. *PcDWF1*, which had a significantly lower level of expression in the dwarf-type pear than in the standard-type pear, was cloned for further analysis. A phylogenetic analysis showed that *PcDWF1* was a pear brassinosteroid biosynthetic gene that was homologous to *AtDWARF1*. The subcellular localization analysis indicated that PcDWF1 was located in the plasma membrane. Overexpression of *PcDWF1* in tobacco (*Nicotiana tabacum*) or pear (*Pyrus ussuriensis*) plants promoted the growth of the stems, which was caused by a larger cell size and more developed xylem than those in the control plants, and the rooting ability was significantly enhanced. In addition to the change in vegetative growth, the tobacco plants overexpressing *PcDWF1* also had a delayed flowering time and larger seed size than did the control tobacco plants. These phenotypes were considered to result from the higher BL contents in the transgenic lines than in the control tobacco and pear plants.

**Conclusions:**

Taken together, these results reveal that the pear BR biosynthetic gene *PcDWF1* affected the vegetative and reproductive growth of *Pyrus ussuriensis* and *Nicotiana tabacum* and could be characterized as an important BR biosynthetic gene in perennial woody fruit plants.

## Background

The plant steroidal hormones brassinosteroids (BRs) are ubiquitously distributed throughout the plant kingdom [1–5]. BRs are involved in the regulation of multiple developmental and physiological processes that are essential for plant vegetative and reproductive growth [6–10]. Though the effects of BR on plant development were known as early as the 1970s, recent molecular genetic studies of BR-deficient and BR-insensitive mutants have established that BRs have an essential role in plant growth and development [11–13].

The deficiency mutants in BR biosynthesis or signaling usually show a characteristic pleiotropic phenotype, including dwarfism, photomorphogenesis in the dark, delayed senescence, and reduced apical dominance and fertility [14–16]. In *Arabidopsis* and rice, the mutants *dwf3*, *dwf4*, and *br6ox1*, which have defects in brassinosteroid biosynthetic genes, all exhibited a conspicuous dwarf phenotype, and this phenotype could be restored by the exogenous application of brassinolide (BL) [17–22]. In the woody plant apple, the roles of BRs in plant growth were preliminarily reported. Ma et al. (2016) reported that dwarfism resulted from the reduced expression of the BR biosynthesis gene *MdDWF4* coupled with the high expression of *BRI1 kinase inhibitor 1* (*MdBKI1*) and *brassinosteroid insensitive 2* (*MdBIN2*) [23, 24]. The molecular genetic studies of BR function were mainly based on key genes in the BR biosynthetic pathway.

Among more than 50 isolated and identified BRs, brassinolide (BL) is the most active one. The BL biosynthetic pathway has been identified primarily through the characterization of BR-deficient mutants in the model plants *Arabidopsis*, *Oryza sativa* (rice), and *Solanum lycopersicum* (tomato) [25]. The BR-specific biosynthetic precursor campesterol (CR) is converted to brassinolide (BL) mainly via four pathways, including the early and late C-6 oxidation pathways, the early C-22 oxidation branch and C-23 hydroxylation shortcuts [26–31]. CR, as the precursor of BL, was conserved from 24-methylenecholesterol (24-MCHR) by a sterol C-24 reductase named DWARF1 (DWF1). DWF1 is also considered to be an important enzyme in the BR biosynthetic pathway [32, 33]. However, the BR biosynthetic pathway and the rate-limiting genes have mainly been studied in herbaceous plants, but still unclear in woody plants [34, 35].

DWF1, as one of the important enzymes in the BR biosynthetic pathway, is a flavin adenine dinucleotide (FAD)-dependent oxidoreductase and Ca^2+^-dependent calmodulin-binding protein [32, 33]. DWF1 is an integral membrane protein, and it is expressed in most of the tissue types in seedlings and sections of the inflorescence stem and predominantly localizes in the xylem vessels and in the interfascicular cambium [36, 37]. A T-DNA mutant of this gene in *Arabidopsis*, *dwf1*, had a significantly dwarfed phenotype, and both its growth and development in the dark and light were significantly retarded [31, 38–41].. Compared to the mutant, the overexpression of *AtDWF1* could slightly increased stem elongation and silique formation and altered root development [40]. However, the function of DWF1 in woody plants has not been explored. In addition, although DWF1 appears to be conserved across plant species, its function is not conserved across species. The *Arabidopsis* and pea *dwf1* knockout mutant showed a severe dwarf phenotype, whereas the rice *dwf1* mutant displayed only a moderate semidwarf phenotype [38, 42]. Therefore, it is of great importance to determine the functions of the homologous genes of DWF1 in woody plants and gain insights into the BR biosynthesis pathway in woody plants.

In this study, we cloned the BR biosynthetic gene *PcDWF1*, which is the homologous gene of *AtDWF1*, from pear*.* A qPCR analysis showed that the expression level of *PcDWF1* was significantly decreased in the dwarf-type pear compared with that in the standard-type pear. A subcellular localization analysis showed that PcDWF1-GFP was located in the plasma membrane. In addition, we overexpressed *PcDWF1* in tobacco and pear and found that the overexpression of *PcDWF1* not only affected the vegetative growth but also the reproductive growth of the transgenic plants. These findings showed the positive role of *PcDWF1* in BR biosynthesis and clarified the function of *PcDWF1* in the vegetative and reproductive growth of woody plants.

## Results

### The effect of exogenous BL on the growth of pears

To elucidate the roles of BL on the growth of pears, *Pyrus ussuriensis* plants in in vitro culture were used. We applied 0 mg/L, 0.1 mg/L, 0.5 mg/L, 1.0 mg/L, and 2.0 mg/L BL to the normal medium of the pear, respectively. After 30 days growth, it was obviously to find that applying with 0.5 mg/L BL could significantly improve the growth of the pear plants, while applying with 0.1 mg/L and 1.0 mg/L BL had fewer effect than 0.5 mg/L BL. When the BL concentration was as high as 2.0 mg/L, the growth of the pear was inhibited (Fig. S1A). The date of plant height and the diameter of the pear stem also supported the phenotype (Fig. S1). So 0.5 mg/L BL was selected for further research.

**Fig. 1:**
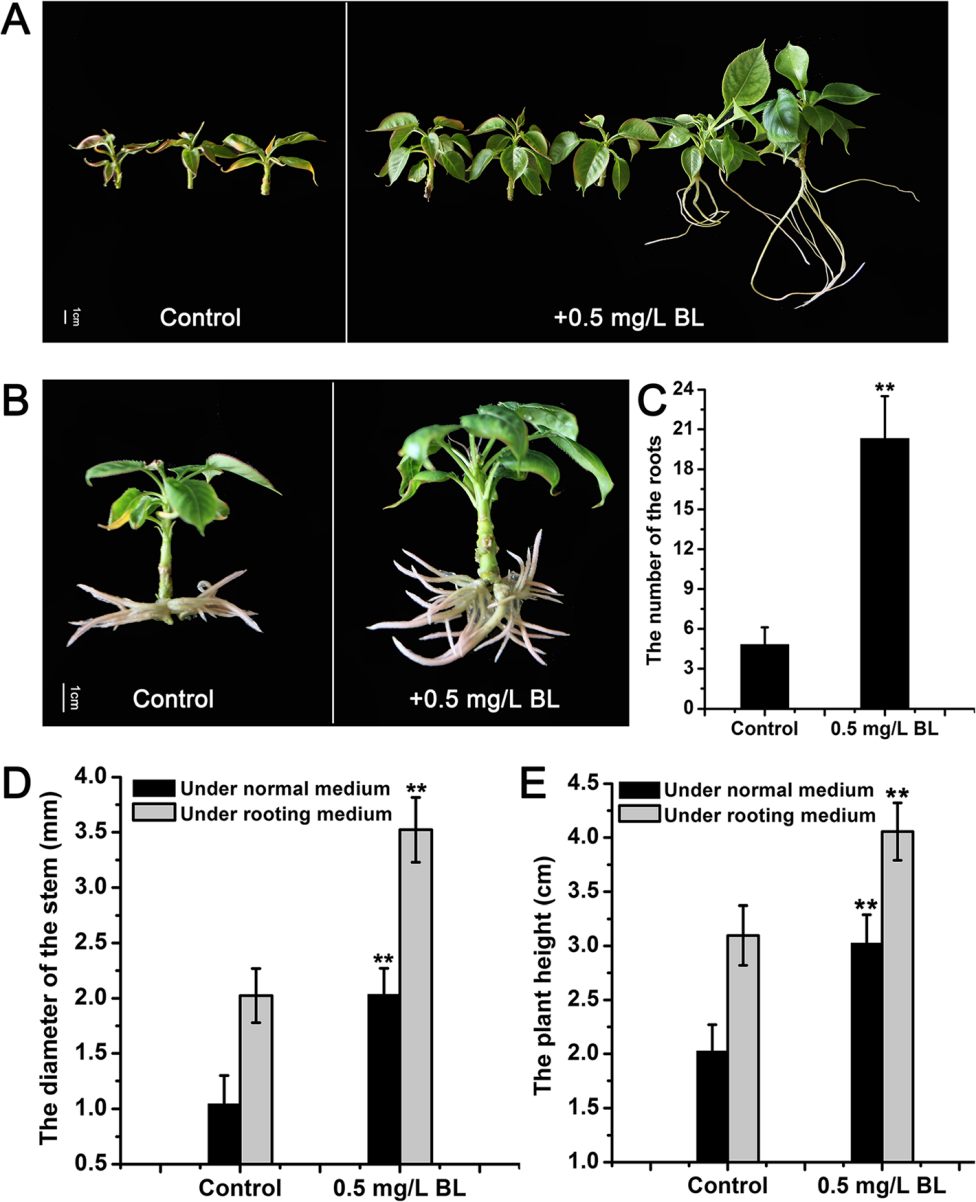
The effect of BL on the growth of *Pyrus ussuriensis*. **a** The phenotypes of *Pyrus ussuriensis* with/without 0.5 mg/L BL treatment on normal MS medium for 30 days. **b** The phenotypes of *Pyrus ussuriensis* with/without 0.5 mg/L BL treatment on rooting medium for 30 days. **c** The number of roots of *Pyrus ussuriensis* with/without 0.5 mg/L BL treatment on rooting medium for 30 days. The diameter of the stem (**d**) and the plant height (**e**) of *Pyrus ussuriensis* with/without 0.5 mg/L BL treatment. Data are the means ± SD of triplicate experiments. Asterisks (*) indicate significant differences from the control (Student’s *t*-test, **P < 0.01)

The pears in the normal medium with 0.5 mg/L BL were much taller and thicker than those in the control medium without BL after 30 days of growth, and some of the pears in the medium with 0.5 mg/L BL were taken roots (Fig. 1a). The height of the pear plants with 0.5 mg/L BL (3.03 cm) was approximately 1.5 times greater than that of the control pear plants (2.02 cm) (Fig. 1e), and the diameter of the pear plants with 0.5 mg/L BL reached 2.03 mm, which was 2 times that of the control pear plants (1.04 mm) (Fig. 1d). For the rooting medium, the pear plants with 0.5 mg/L BL was also much taller and thicker than the control pear after 30 days of growth (Fig. 1b, d, e). In addition, the number of roots increased after applying 0.5 mg/L BL. The average number of roots in the control pear was 5, with only 1/4 the number of the roots in the pear with 0.5 mg/L BL (20) (Fig. 1c). These results indicated that exogenous BL not only increased the diameter and height of the pear plants but also increased the rooting ability of the pear.

### Exogenous BL could partly rescue the dwarf phenotype of the dwarf-type pears

**Fig. 2:**
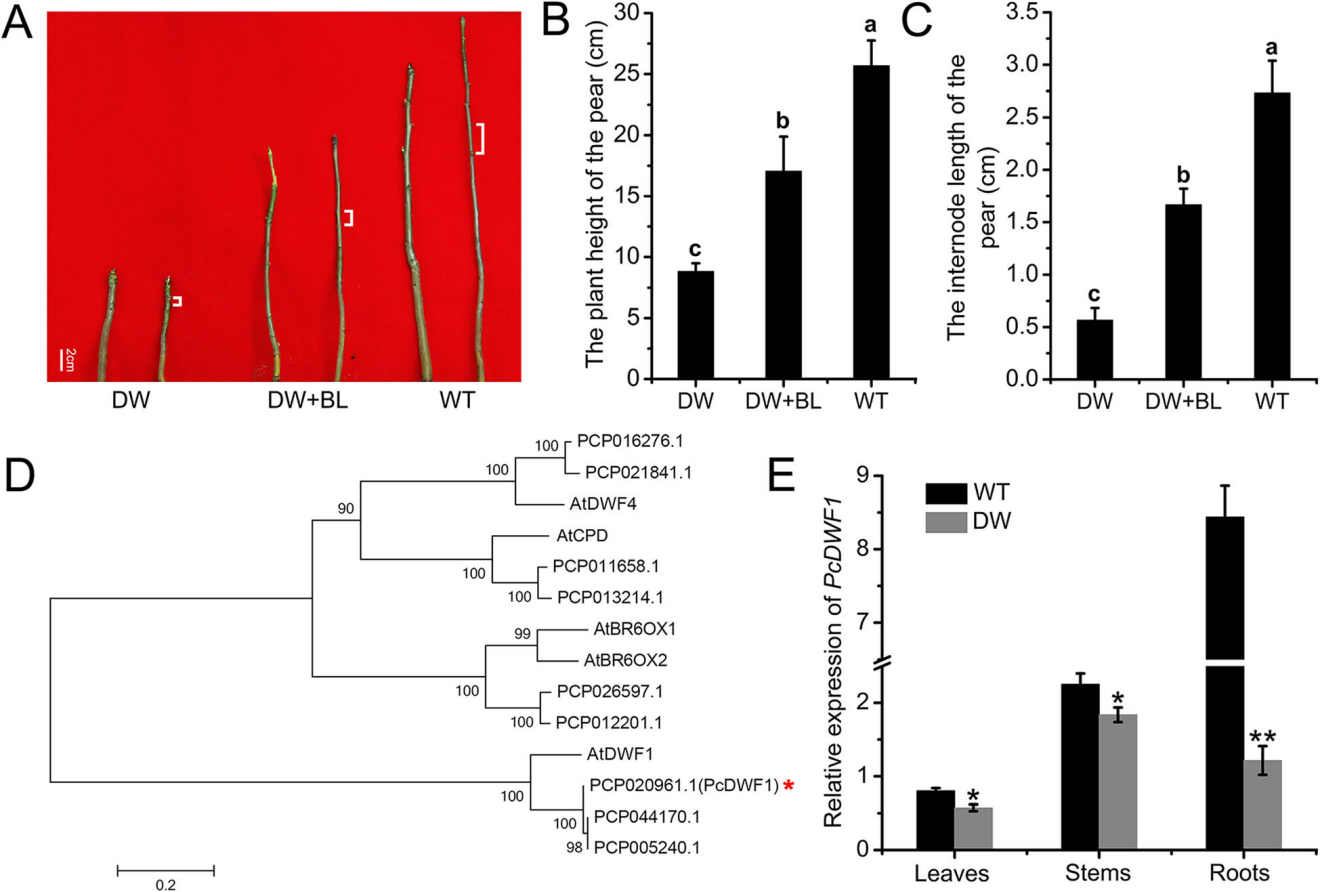
The effect of exogenous BL on the dwarfing phenotype of dwarf-type pears and the expression of *PcDWF1* in different tissues of the dwarf-type (DW) and standard-type (WT) pears. **a** The phenotype of the dwarf-type pear plants after spraying with exogenous BL for 1 month, with the dwarf-type and standard-type pears as the control. The plant height (**b**) and internode length (**c**) of the dwarf-type pears after spraying with exogenous BL for 1 month, with the dwarf-type and standard-type pears as the control. **d** The phylogenetic tree was constructed using MEGA5.2 software with the neighbor-joining method and a bootstrap test with 1000 iterations based on the amino acid sequences of BR biosynthetic rate-limiting genes in pear and *Arabidopsis*. **e** The expression of *PcDWF1* in the leaves, stems, and roots of dwarf-type and standard-type pears. Data are the means ± SD of triplicate experiments. Different lowercase letters indicate significant differences according to Fisher’s LSD (*P* < 0.05). Asterisks (*) indicate significant differences from the control (Student’s *t*-test, *P < 0.05, **P < 0.01)

To see the effect of exogenous BL on the dwarf-type pears, we applied 0.5 mg/L BL to the dwarf-type pears. As shown in Fig. 2a, application with 0.5 mg/L BL could partly recover the dwarf phenotype with longer internode length compared with dwarf-type pears with no BL application. The plant height of the dwarf-type pears with 0.5 mg/L BL was 17.03 cm, about two times of the control dwarf-type pears (8.83 cm) (Fig. 2b). The internode lengths of standard-type pears and dwarf-type pears were 2.73 cm and 0.56 cm, respectively. However, the internode length of the dwarf-type pears with BL treatment was as long as 1.67 cm, two times longer than control dwarf-type pears (Fig. 2c). These results indicated that exogenous BL could affect the stem growth and partly rescue the dwarf phenotype of the dwarf-type pears.

### *PcDWF1*, the BR biosynthetic gene, affected the growth of the pear

To determine the key BR biosynthetic genes that affected the growth of the pear, we determined the key rate-limiting BR synthetic genes in pear (*PCP020961.1*, *PCP044170.1*, *PCP005240.1*, *PCP016276.1*, *PCP021841.1*, *PCP011658.1*, *PCP013214.1*, *PCP026597.1*, and *PCP012201.1*), which are homologous genes of the rate-limiting BR synthetic genes in *Arabidopsis* (*AtDWF1*, *AtCPD*, *AtDWF4*, *AtBR6OX1*, and *AtBR6OX2*) (Fig. S2). A phylogenetic tree based on the amino acid sequences of these genes was constructed using MEGA 5.2 software to see their homologous relationship (Fig. 2d). To determine which BR biosynthetic gene affected plant growth the most, the expression of these genes in the leaves, stems, roots of dwarf-type and standard-type pears were detected. It was found that the expression of *PCP020961.1* decreased significantly in the leaves, stems and roots of the dwarf pears, while the other eight genes had no significant difference (Fig. S3). Specifically, in the roots, the expression of *PCP020961.1* in the dwarf-type pear was only 1/7 of that of the standard-type pear (Fig. 2e). This result indicated that *PCP020961.1* (*PcDWF1*), which is the homologous gene of *AtDWF1*, would take part in the pear growth. The CDS of *PcDWF1* was cloned from the dwarf-type and standard-type pears. As shown in Figure S4A, the CDS sequence of *PcDWF1* had no difference between the dwarf-type and standard-type pears. In addition, we also detected the expression level of *PcDWF1* under exogenous BL treatment to the *Pyrus ussuriensis* plants. The expression of *PcDWF1* was significantly decreased by exogenous BL treatment no matter in normal medium or in rooting medium (Fig. S4B). These combined results indicated that *PcDWF1* might take part in the BR biosynthetic pathway and involving in the growth of the pear.

### PcDWF1 was located in the plasma membrane

**Fig. 3:**
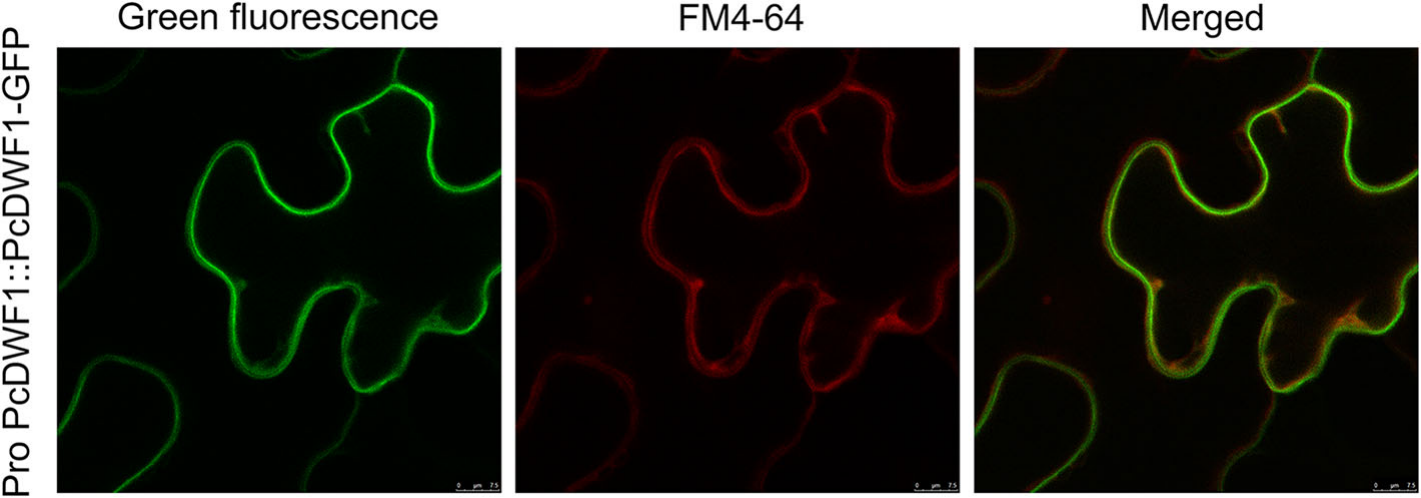
Subcellular localization of PcDWF1. PcDWF1-GFP was located in the plasma membrane of *Nicotiana benthamiana* cells. FM4–64 was a marker of plasma membrane

To determine the location of PcDWF1, the CDS without the stop codon of *PcDWF1* and the promoter of *PcDWF1* were ligated into *pMDC83* vector to generate a *Pro PcDWF1::PcDWF1-GFP* construct. To examine the subcellular localization of PcDWF1-GFP, GV3101 harboring *Pro PcDWF1::PcDWF1-GFP* was introduced into *Nicotiana benthamiana* leaves by *Agrobacterium*-mediated transient transformation. Three days later, PcDWF1-GFP fluorescence was observed overlaps with plasma membrane marker FM4–64. This result indicated that PcDWF1 was located in the plasma membrane (Fig. 3).

### Phenotype of the transgenic tobacco lines overexpressing *PcDWF1*

**Fig. 4:**
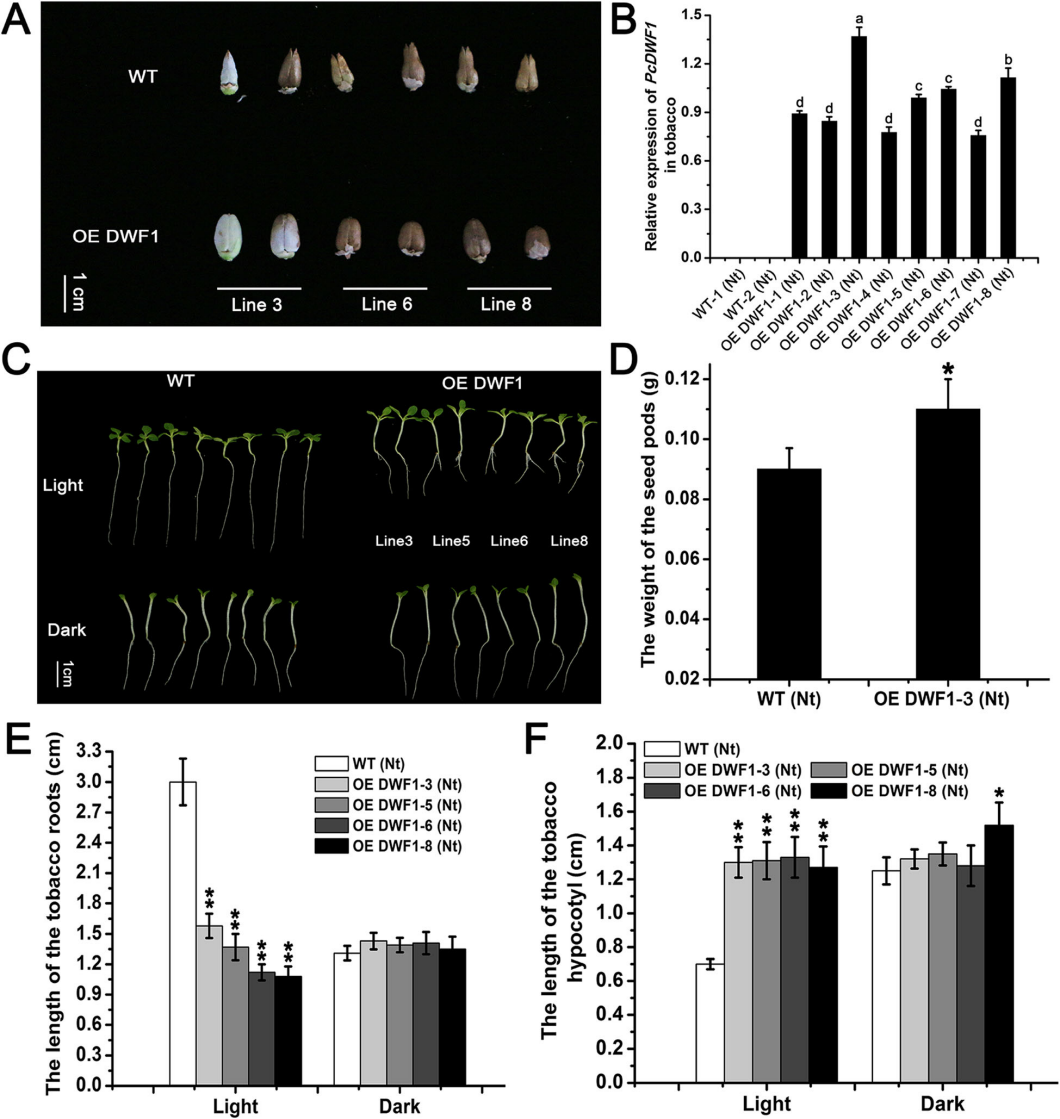
The phenotypes of the seeds and the seedlings of the transgenic lines overexpressing *PcDWF1* and the control tobacco plants. **a** The seeds of the transgenic lines overexpressing *PcDWF1* and the control tobacco plants. **b** The expression of *PcDWF1* in eight transgenic tobacco lines and the control tobacco plants. **c** The phenotypes of the transgenic tobacco lines and the control tobacco seedlings under continuous light/dark conditions for 10 days. **d** The weight of the seed pods of the transgenic tobacco line 3 and the control tobacco plants. The root length (**e**) and the hypocotyl length (**f**) of the transgenic tobacco lines and the control tobacco seedlings under continuous light/dark conditions for 10 days. Data are the means ± SD of triplicate experiments. Different lowercase letters indicate significant differences according to Fisher’s LSD (*P* < 0.05). Asterisks (*) indicate significant differences from the control (Student’s *t*-test, *P < 0.05, **P < 0.01)

To investigate the function of *PcDWF1* in the regulation of growth, we overexpressed *PcDWF1* in tobacco. A total of eight transgenic lines were achieved, and the expression of *PcDWF1* in lines 3, 5, 6, and 8 was much higher than that in the wild type. Therefore, these four lines were selected for further analysis (Fig. 4b).

The seeds of these transgenic lines were larger and more plump than the wild type seeds (Fig. 4a). The seed pods of the transgenic tobacco line 3 (0.11 g) were significantly heavier than the wild type seed pods (0.09 g) (Fig. 4d). We also found that the growth of the transgenic tobacco lines overexpressing *PcDWF1* and the wild type tobacco seedlings was affected by light. When the plants were kept in the dark for 10 days, the lengths of the hypocotyls and the roots of the transgenic lines were not significantly different from those in the wild type. Under continuous light for 10 days, the hypocotyl lengths of the transgenic lines were much greater than those of the control tobacco plants, and the transgenic plants had clear lateral roots. The hypocotyl length of line 6 reached 1.33 cm, which was approximately 2 times that of the wild type (0.7 cm) (Fig. 4c, f). In addition, the roots of the transgenic lines were much shorter than those of the control tobacco plants, and the root length of line 8 was only 1.08 cm, while that of the wild type was nearly 3 cm (Fig. 4c, e). We also detected the *PcDWF1* gene expression and BL contents under continuous light and dark treatment. It was found that the expression level of *PcDWF1* was significantly higher under continuous light treatment than that under continuous dark treatment (Fig. S5A). It was also interested to find that the BL contents of the transgenic tobacco lines were significantly higher than that in wild type tobacco plants under continuous light treatment, while there was no significantly difference under continuous dark treatment (Fig. S5B).

**Fig. 5:**
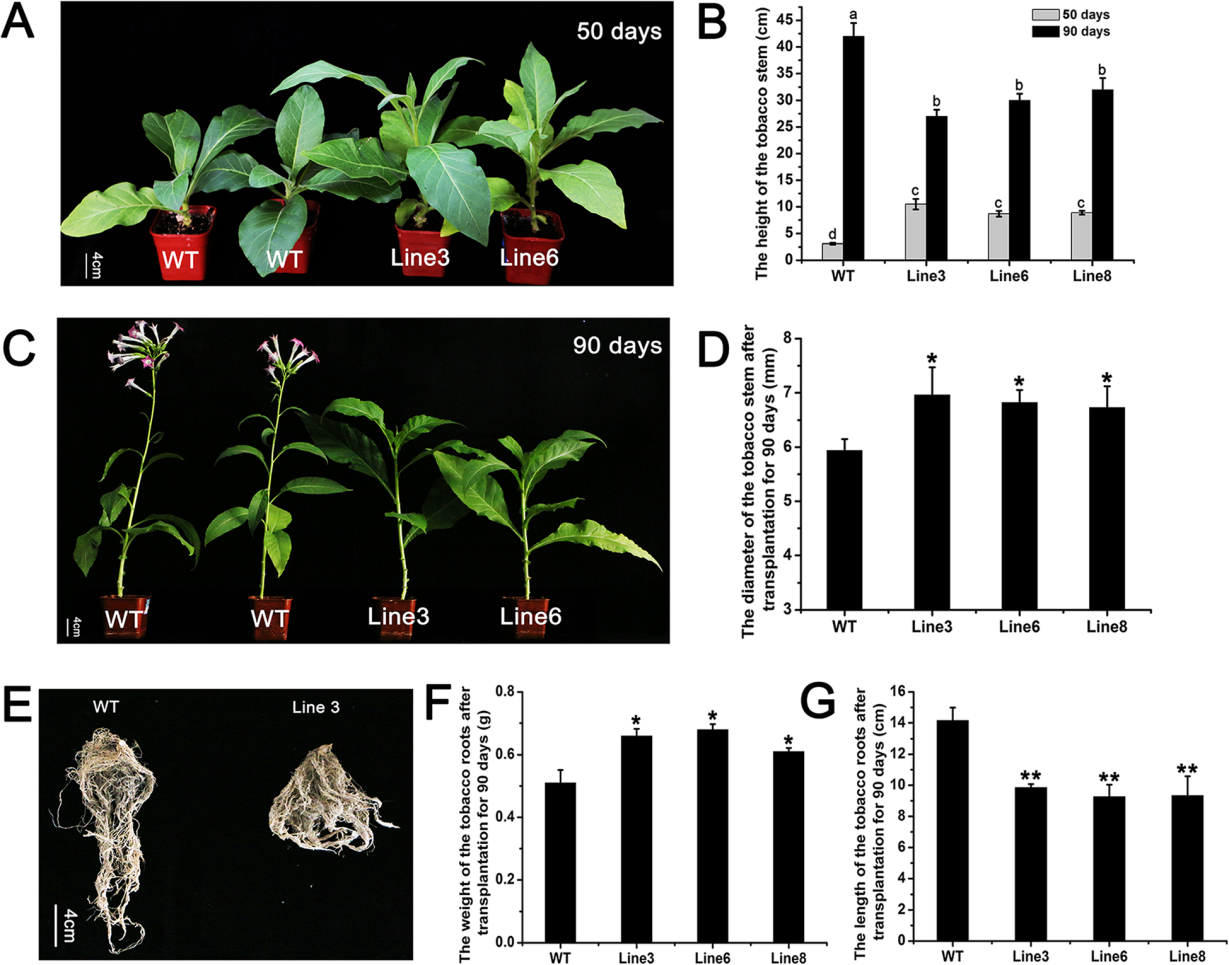
The phenotypes of the transgenic lines overexpressing *PcDWF1* and the control tobacco plants in soil. **a** The transgenic lines overexpressing *PcDWF1* and the control tobacco plants grown under greenhouse conditions after transplantation for 50 days (A) and 90 days (C). **b** The stem height of the transgenic lines overexpressing *PcDWF1* and the control tobacco plants at 50 and 90 days. **d** The stem diameter of the transgenic lines overexpressing *PcDWF1* and the control tobacco plants at 90 days. **e** The root phenotypes of the transgenic lines overexpressing *PcDWF1* and the control tobacco plants at 90 days. The root weight (**f**) and length (**g**) of the transgenic lines overexpressing *PcDWF1* and the control tobacco plants at 90 days. Data are the means ± SD of triplicate experiments. Different lowercase letters indicate significant differences according to Fisher’s LSD (*P* < 0.05). Asterisks (*) indicate significant differences from the control (Student’s *t*-test, *P < 0.05, **P < 0.01)

After the transgenic tobacco lines and the wild type were transplanted into soil for 50 days of growth, it was found that the transgenic lines were significantly taller than the wild type (Fig. 5a). The height of the wild type was only 3.1 cm, but the height of transgenic line 3 reached 10.5 cm (Fig. 5b). However, the height of the wild type was greater than that of the transgenic lines after 90 days (Fig. 5c). At this time, the height of the wild type was 42 cm, which was much higher than that of transgenic line 3 (27 cm) (Fig. 5b). The diameter of the stem was also measured, and it was found that the transgenic lines had thicker stems than did the wild type. The diameter of the wild type stems was 5.94 mm, which was significantly thinner than those of transgenic line 3 (6.96 mm) and line 6 (6.82 mm) (Fig. 5d). In addition, it was also interesting to find that the flowering time of the transgenic lines was delayed by approximately 10 days compared to that of the wild type (Fig. 5c). In the underground part of the plants, the transgenic lines had more but shorter roots than the wild type (Fig. 5e). For instance, the root weight of transgenic line 6 (0.65 g) was significantly greater than that of the wild type (0.51 g) (Fig. 5f), while the root length of line 6 (9.26 cm) was only 2/3 of that of the wild type (14.15 cm) (Fig. 5g). These results indicated that overexpressing *PcDWF1* changed the growth of both the aboveground part and the underground parts of tobacco plants.

### Phenotype of the transgenic pear lines overexpressing *PcDWF1*

**Fig. 6:**
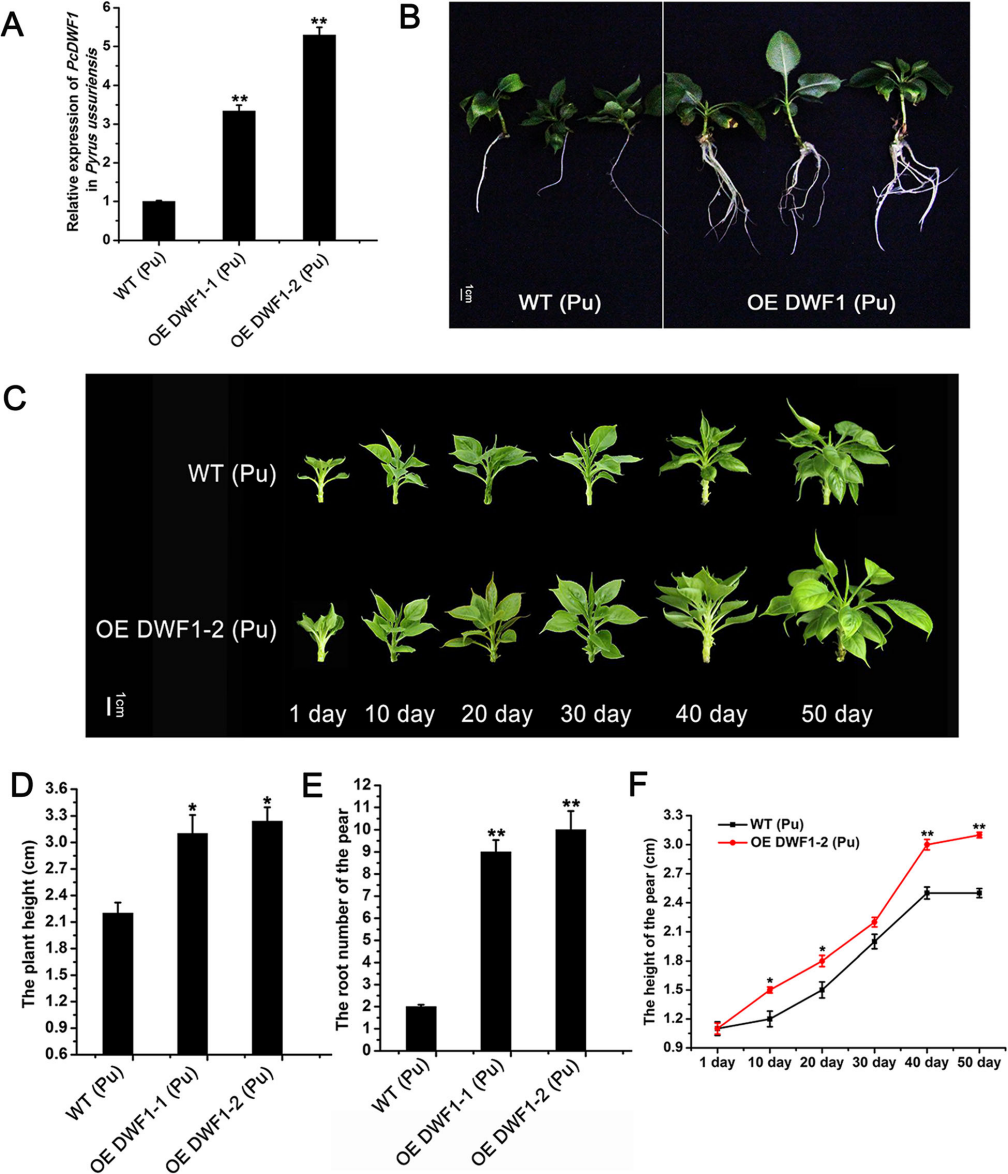
The phenotypes of the transgenic pear lines overexpressing *PcDWF1* and control pear plants. **a** The expression of *PcDWF1* in the transgenic pear lines and control pear plants. **b** The phenotypes of the transgenic pear lines and the control pear plants on rooting medium for 50 days. **c** The growth state of the transgenic pear lines and the control pear plants on subculture medium for 50 days. The plant height (**d**) and the root number (**e**) of the transgenic pear lines and the control pear plants on rooting medium for 50 days. (**f**) The pear height changes in the transgenic pear lines and control pear plants on subculture medium in 50 days. Data are the means ± SD of triplicate experiments. Asterisks (*) indicate significant differences from the control (Student’s *t*-test, *P < 0.05, **P < 0.01)

To investigate the function of *PcDWF1* in the regulation of pear growth, we overexpressed *PcDWF1* in *Pyrus ussuriensis*, and two transgenic lines (line 1 and line 2) were generated. The qPCR results showed that the expression level of *PcDWF1* in line 1 was 3 times that of the wild type, while the expression level of *PcDWF1* in line 2 was 5 times that of the wild type (Fig. 6a). The transgenic seedlings and the wild type seedlings were cultured in normal MS medium and rooting medium. For the rooting medium, the transgenic lines were much taller and had more roots than the wild type after 50 days of growth (Fig. 6b). The height of the wild type was 2.2 cm, while that of line 1 was 3.1 cm and line 2 was 3.24 cm (Fig. 6d). The root number of line 2 was 10, which was 5 times greater than that of the wild type (2) (Fig. 6e). For the normal subculture medium, the growth progress was recorded at 10-day intervals until 50 days. As clearly shown in Fig. 6c, the growth rate of transgenic line 2 was much faster than that of the wild type. The height of line 2 was significantly greater than that of the wild type at day 10 and day 20, and then the wild type grew faster than line 2 at day 30. Lastly, the height of line 2 reached as high as 3.1 cm at day 50, and it was significantly higher than that of the wild type (2.5 cm) (Fig. 6f).

We also detected the expression of *PcDWF1* downstream genes of the BR biosynthetic pathway (*PcCPD-1*, *PcCPD-2*, *PcDWF4–1*, *PcDWF4–2*, *PcBR6OX1–1*, and *PcBR6OX1–2*) in transgenic and wild type pear plants. It was found that the expression of *PcCPD-1*, *PcDWF4–2*, and *PcBR6OX1–1* were significantly induced in transgenic pear plants, while the other three genes had no significantly difference (Fig. S6). This result indicated that *PcDWF1*, *PcCPD-1*, *PcDWF4–2*, and *PcBR6OX1–1* might be the key BR biosynthetic genes in pear.

### The effect of *PcDWF1* overexpression on the BL content

**Fig. 7:**
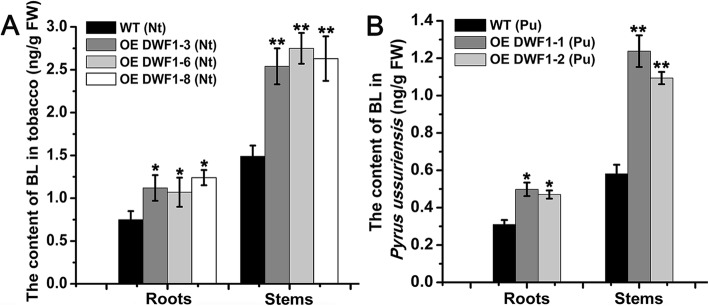
The BL contents of the roots and stems of the transgenic lines overexpressing *PcDWF1* and the control tobacco and pear plants. Data are the means ± SD of triplicate experiments. Asterisks (*) indicate significant differences from the control (Student’s t-test, *P < 0.05, **P < 0.01)

*DWF1* is the key gene in BL biosynthesis. The BL contents in the leaves, stems, and roots of the transgenic lines and wild type tobacco and pear plants were determined. As shown in Fig. 7a, the BL contents were significantly increased in the roots and stems of the transgenic lines, while the BL contents were not detected in leaves. The most significant change was in the stem; the BL content in the wild type was 1.49 ng/g, but that in transgenic line 6 and line 8 was 2.75 ng/g and 2.63 ng/g, respectively (Fig. 7a). The BL contents of the transgenic pear lines were also significantly higher than that of wild type pears in both roots and stems (Fig. 7b). These results indicated that overexpressing *PcDWF1* could increase the BL content in both tobacco and pear plants.

### The comparison of anatomical structures between the transgenic lines and wild type tobacco and pear plants

**Fig. 8:**
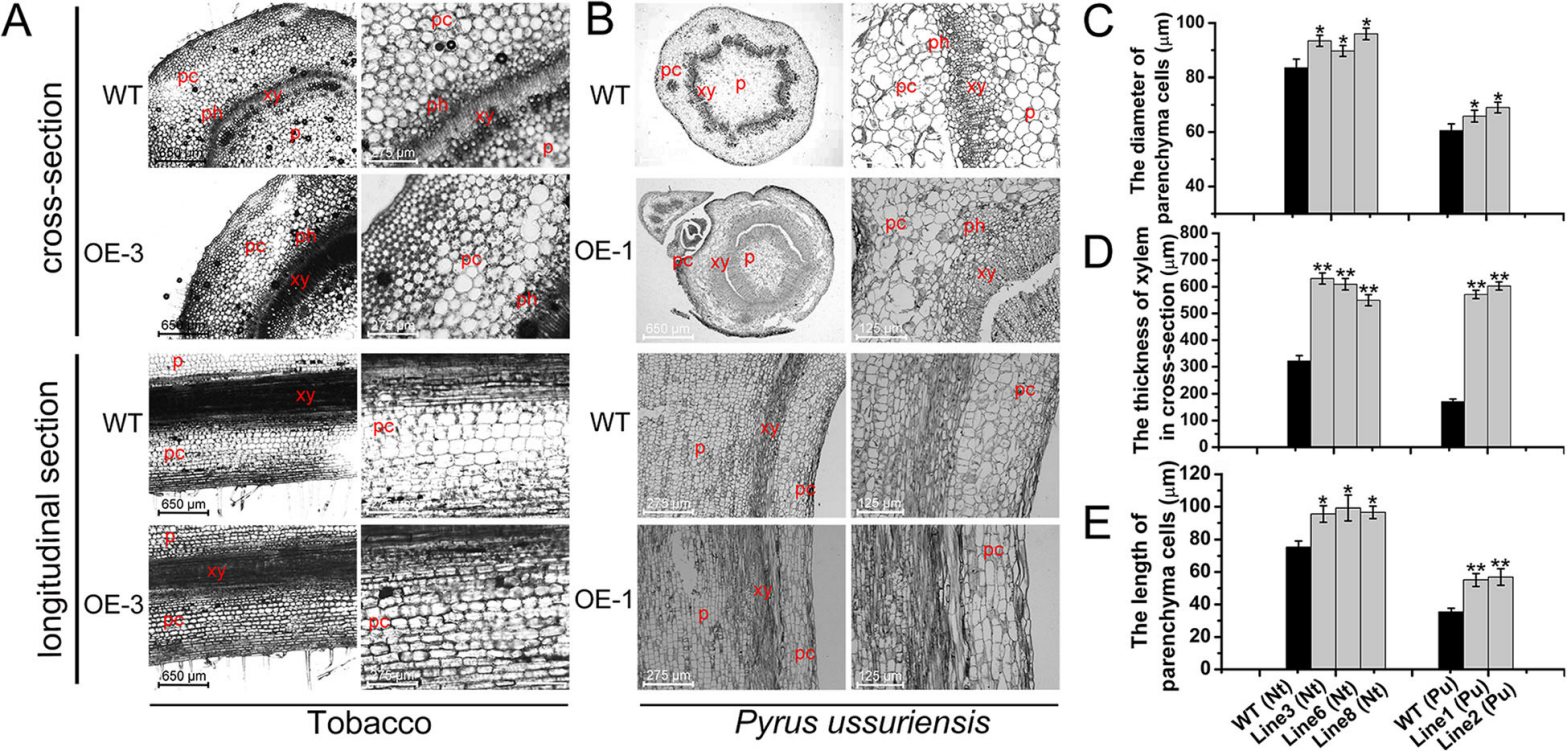
Anatomical structure analysis of the transgenic lines and the control tobacco and pear plants, including the cross-section and the longitudinal section of the stems. **a** The anatomical structure analysis of the transgenic lines and the control tobacco plants. pc, parenchymal cell; xy, xylem; ph, phloem; p, pith. **b** The anatomical structure analysis of the transgenic lines and the control pear plants. **c** The diameter of the parenchymal cells in the stems of transgenic lines and the control tobacco and pear plants. **d** The thickness of the xylem of transgenic lines and the control tobacco and pear plants in cross-section. **e** The length of the parenchymal cells in the stems of transgenic lines and the control tobacco and pear plants in longitudinal section. Data are the means ± SD of triplicate experiments. Asterisks (*) indicate significant differences from the control (Student’s t-test, *P < 0.05, **P < 0.01)

To determine the potential role of *PcDWF1* in plant growth and development, the differences in the anatomical structures of the stems between the transgenic lines and control pear and tobacco plants were observed. In the cross-section, it was clear that the cell size of the parenchymal cells in transgenic tobacco line 3 were much larger than those of the control tobacco (Fig. 8a, c). Another significant change was the xylem, and the xylem of the transgenic lines was much thicker than that of the wild type tobacco (Fig. 8a, d). Similar result was also observed in pear. The diameter of the parenchymal cells and the thickness of xylem in the transgenic pear lines were significantly bigger than that in wild type pears (Fig. 8). These results indicated that the transgenic lines overexpressing *PcDWF1* increased the diameter of their stems primarily through enhancing cell size of the parenchymal cells and the xylem.

In longitudinal section, the parenchymal cells of the transgenic lines overexpressing *PcDWF1* were much longer and denser than those in the wild type pear and tobacco plants (Fig. 8). These results showed that the transgenic lines overexpressing *PcDWF1* increased their stem height mainly through promoting cell division and cell elongation in the parenchymal cells.

## Discussion

Brassinosteroids (BRs) are a group of polyhydroxylated steroidal phytohormones that play critical roles in multiple processes of plant vegetative and reproductive growth, such as stem elongation, flowering time, vascular differentiation, photomorphogenesis, root development, and responses to various stresses [43–45]. Among these various functions, the pivotal role of BRs is the regulation of plant growth [46]. Many studies have reported that the application of exogenous BL could affect the growth of plants. In soybean, applying exogenous BL could promote stem growth and adventitious rooting and increase soybean tolerance to drought stress [47–49]. In the woody plant apple, it was reported that exogenous BL could promote stem growth and increase lateral root development in *Malus hupehensis* seedlings [50]. In our study, to observe the effect of exogenous BL on the growth of pears, we added 0.5 mg/L BL to normal MS medium and rooting medium (1/2 MS) for *Pyrus ussuriensis.* After 30 days of growth, the results indicated that the exogenous BL could not only improve the growth of the stem but could also enhance the rooting ability of the pear (Fig. 1). This was the first report on the function of exogenous BL in pear seedlings in in vitro culture. In addition, our result also indicated that applying 0.5 mg/L exogenous BL could partly rescue the dwarfing phenotype with longer internode length of the dwarf-type pears (Fig. 2), further conforming its role in the stem growth of the pear.

A previous study found that brassinosteroid (BR) is involved in many different biological processes, as reflected by the expression of BR biosynthetic genes [51]. To determine which BR biosynthetic genes play the most important roles in plant growth and development in pear, we isolated nine pear homologous genes of the important BR biosynthetic genes in *Arabidopsis* (*AtDWF1*, *AtCPD*, *AtDWF4*, *AtBR6OX1*, and *AtBR6OX2*) [15, 20, 21] (Fig. S2). The expression of the nine genes was detected in dwarf-type and standard-type pears (Fig. S3), which were derived from the cross of dwarf pear ‘Aiganlu’ and standard pear ‘Zhongxiangli’. The results showed that the expression of *PcDWF1*, which is the homologous gene of *AtDWF1*, was significantly lower in dwarf-type pears than in standard-type pears in the roots, stems and leaves (Fig. 2). In addition, we also found that the expression of *PcDWF1* was significantly decreased by exogenous BL treatment, indicating that PcDWF1 might be involved in BR biosynthetic pathway (Fig. S4). Therefore, *PcDWF1* was considered one of the important BR biosynthetic genes affecting pear plant growth.

To further explore the fundamental characteristics and functions of *PcDWF1*, we cloned the CDS domain of *PcDWF1* from the dwarf-type and standard-type pears, and the result showed no difference in dwarf-type and standard-type pears (Fig. S4). To determine the subcellular location of PcDWF1, the coding sequence without the stop codon of *PcDWF1* was fused with GFP and drived by its nature promoter. After 3 days of transient transformation in *Nicotiana benthamiana*, the PcDWF1-GFP proteins were observed overlaps with plasma membrane marker FM4–64 (Fig. 3). PcDWF1 shares a very high amino acid sequence identity with *Arabidopsis* AtDWF1. In 1998, Klahre also detected the subcellular location of AtDWF1 in germinating pollen tubes, and the AtDWF1-GFP fusion protein was localized in a speckled pattern in a compartment in the cytosol, which was most likely the ER. In addition, cell fractionation studies have shown that AtDWF1 is an integral membrane protein [36]. Our results were in agreement with that DWF1 was an integral membrane protein with the subcellular localization of PcDWF1-GFP was located in plasma membrane. In our opinion, these differences may be caused by the localization of DWF1 in different tissues (pollen tube/leaves), and this result needs further investigation.

To further analyze the function of *PcDWF1* in pear growth and development, we overexpressed *PcDWF1* in tobacco and *Pyrus ussuriensis.* The transgenic lines with a high expression level of *PcDWF1* were selected for further analysis. As shown in Fig. 5a, the transgenic tobacco lines all showed significantly taller and thicker stems than did the wild type after they were transplanted into soil for 50 days. A similar phenotype of the transgenic pear was observed in both the normal MS medium and rooting medium (Fig. 6). These phenotypic alterations are consistent with those in previous reports in transgenic *Arabidopsis* overexpressing *AtDWF1*, which displayed clearly elongated and thickened stems [37, 40]. To further analyze the cause of the fast growth of the stem, we observed the anatomical structure of the transgenic lines overexpressing *PcDWF1* and the wild type tobacco and pear plants. The results showed that the cell size of the parenchymal cells and the xylem of the stem in the transgenic lines were much larger and more developed than those in the control pear and tobacco plants. In addition, the cells in the parenchymal cells of the transgenic lines overexpressing *PcDWF1* were longer and denser than those in the wild type tobacco and pear plants (Fig. 8). The xylem functions to transport water upward, and a more developed xylem is beneficial for the growth of the stem [52, 53]. These results indicated that the thicker stem of the transgenic lines was mainly caused by a larger cell size and the increased stem height was mainly caused by elongated cells in the parenchymal cells and a more highly developed xylem. Extensive evidence has shown that BR plays important roles in cell expansion and xylogenesis [25, 53]. Moreover, previous research has shown that the dwarf phenotype of BR-deficient and insensitive mutants mainly resulted from a reduction in cell size rather than a reduction in cell number [16]. We hypothesized that these phenotype similarities are probably due to the altered activity of the BR biosynthetic enzyme, thus influencing BR biosynthesis in tobacco and pear. To test this hypothesis, the contents of BL in the transgenic tobacco lines overexpressing *PcDWF1* and the control tobacco and pear plants were detected. The results showed that the BL contents in the stems of transgenic lines were significantly higher than that in the stems of the control pear and tobacco plants (Fig. 7). Taken together, the taller and thicker stems of the transgenic tobacco and pear plants overexpressing *PcDWF1* were mainly caused by the greater cell division and cell elongation, larger cell size, and a more developed xylem, which resulted from the higher BL content in the transgenic stems than in the control stems. In plants overexpressing BR, synthesis-related genes could enhance the height and diameter of the stem [54]. These results also suggested that *PcDWF1* was an important BR biosynthetic gene in pear. Moreover, our result also showed that the expression of *PcCPD-1*, *PcDWF4–2*, and *PcBR6OX1–1* were significantly induced in transgenic pear plants overexpressing *PcDWF1* (Fig. S6). This result indicated that *PcDWF1*, *PcCPD-1*, *PcDWF4–2*, and *PcBR6OX1–1* might be the key BR biosynthetic genes in pear.

In the underground part of the plants, the roots were obviously different between the transgenic lines overexpressing *PcDWF1* and the control tobacco/pear plants. In tobacco, the root length of the transgenic lines was shorter than that in the wild type. However, the weight of the roots and the root number of the transgenic tobacco lines were significantly greater than those in the control tobacco plants (Fig. 5). A similar root phenotype was observed in the transgenic pear, and the root number of the transgenic pear was much higher than that of the control pear after growing on rooting medium for 50 days (Fig. 6). These root phenotypes are typical of changes in BR. BR has been reported to have a positive effect on lateral root development. Seedlings grown in MS medium containing low concentrations of BL (1–100 nM) exhibited an increased number of lateral roots in *Arabidopsis* [55]. BR has also been demonstrated to play an important regulatory role in lateral root development in apple and other species [50, 51, 56]. In our study, we detected the BL contents in the roots of the transgenic lines and the control tobacco and pear plants. As shown in Fig. 7, the BL contents in the roots of the transgenic lines overexpressing *PcDWF1* was significantly higher than that in the roots of the wild type tobacco and pear plants, indicating that the root phenotype may be caused by a higher BL content.

BR is involved in the regulation of hypocotyl elongation and photomorphogenesis, and previous studies have analyzed the crosstalk between light and BR. In our research, when the transgenic tobacco seedlings overexpressing *PcDWF1* and the control tobacco seedlings were grown under continuous light for 10 days, the hypocotyls of all the transgenic lines were significantly elongated, and the primary roots were shortened relative to the root length of the control tobacco seedlings (Fig. 4). In addition, we also found that the BL contents were significantly increased in the transgenic tobacco plants overexpressing *PcDWF1* under continuous light treatment (Fig. S5). These results indicated that BR can promote hypocotyl elongation and root formation in light, and these results were in agreement with those from previous studies in *Arabidopsis*. The seedlings of a T-DNA mutant of AtDWF1, *dim*, had shorter hypocotyls than did the wild type seedlings in light [38, 57]. In addition, mutants deficient in BR response and synthesis genes, such as *cpd*, *det2*, *dwf1*, *dwf4*, *bri1*, all showed a short hypocotyl phenotype in light [58, 59]. However, many of the findings of BR-related studies have focused on hypocotyl growth in darkness. Zhang reported that BR was required to accumulate sugar for hypocotyl elongation in *Arabidopsis* under dark conditions [2]. Here, when the plants were grown under continuous darkness for 10 days, the hypocotyls and the roots of the transgenic tobacco were not different from those of the control tobacco plants, and the BL contents of the transgenic tobacco lines also had no significant difference with wild type tobacco plants (Figs. 4, S5). These results indicated that PcDWF1 may mainly take part in photomorphogenesis but not in skotomorphogenesis, as no change in hypocotyl growth was seen between the transgenic and wild type plants in the dark.

In addition to the change in vegetative growth, the overexpression of *PcDWF1* also affected the reproductive growth of tobacco plants. In our study, the flowering time of the transgenic lines overexpressing *PcDWF1* was noticeably delayed by 10 days compared with that of the control tobacco plants. Compared to previous studies, it was interesting to find that overexpressing the different BR biosynthetic genes in *Arabidopsis* resulted in different flowering times relative to that of the wild type. The flowering time of plants overexpressing *PeDWF4* was clearly advanced and that of plants overexpressing *PeCPD* was obviously delayed relative to the flowering time of the wild type in *Arabidopsis thaliana* [60, 61]. The flowering times of the BR-deficient mutants *det2* and *dwf4* and the BR signal receptor mutant *bri1* were all delayed compared with that of the wild type [62]. These findings indicated that BRs play a critical role in regulating the flowering time of plants, but the molecular mechanism needs to be further explored. We hypothesized that the delay in flowering time caused by overexpressing *PcDWF1* is possibly due to an increase in vegetative growth time, with more nutrients accumulating to increase the plant height and diameter and leading to a delay in the transition from vegetative growth to reproductive growth. In addition, seed development in flowering plants is also affected by BR. An important role of BR in plant seed development has been suggested by studies of BR-deficient mutants of *Arabidopsis*, tomato, and rice [46, 63, 64]. Rice mutants with defects in BR biosynthesis or signaling showed a reduced seed length [65]. The overexpression of a BR biosynthetic gene in rice increased seed filling and seed size [66, 67]. In our study, as clearly seen in Fig. 4, the seed size of the transgenic lines overexpressing *PcDWF1* was significantly larger than that of the control tobacco plants, and the seeds were heavier than those in wild type tobacco. Taken together, the results showed that the overexpression of *PcDWF1* in tobacco resulted in an increased BL content, which affected the reproductive growth of the tobacco plants.

## Conclusions

In conclusion, we found that exogenous BL could promote pear stem and root growth. *PcDWF1*, which is as an important BR biosynthetic gene in pear, had a significantly lower level of expression in dwarf-type pear than in standard-type pear. We overexpressed *PcDWF1* in tobacco and pear and found that the transgenic lines had longer and thicker stems with larger cells and increased xylem formation than did the wild type plants, and these changes were caused by the higher BL content in the transgenic lines. The transgenic lines overexpressing *PcDWF1* also had stronger rooting ability than did the wild type. In addition, the overexpression of *PcDWF1* also affected reproductive growth. This study can help us to determine the biosynthesis of BR in pear, which can be applied in molecular breeding efforts to produce improved dwarf cultivars that meet the demands of modern pear production.

## Methods

### Plant materials and growth conditions

For the subculture of the *Pyrus ussuriensis* plants [which were provided from Prof. Yang’s lab (Qingdao Agricultural University)], they were cultured on Murashige and Skoog (Duchefa, Haarlem, Netherlands) medium containing 0.1 mg/L indole-3-butyric acid (IBA) and 1 mg/L 6-benzylaminopurine (6-BA), and they were transferred to fresh medium once a month. For the rooting culture of the *Pyrus ussuriensis* plants, they were cultured on ½ MS medium containing 0.5 mg/L IBA and rooted 1 month later. The *Pyrus ussuriensis* plants were grown under a 16/8 h light/dark cycle at 23 ± 2 °C with a light intensity of 80 μmol·m^− 2^·s^− 1^.

For the dwarf-type and the standard-type pears, they were from the population produced by the dwarf pear ‘Aiganlu’, whose dwarf trait originated from the French dwarf pear variety ‘Le Nain Vert’ (*Pyrus communis* L.), crossed with the Chinese standard pear variety ‘Zhongxiangli’ (*Pyrus bretschneideri* Rehd.). The seedlings of the dwarf-type and the standard-type pear were grown in soil under a 16/8 h light/dark cycle at 23 ± 2 °C with a light intensity of 100 μmol·m^− 2^·s^− 1^ and a relative humidity of 60%.

The seeds of the wild type (which were provided from Prof. Yang’s lab) and transgenic tobacco lines ectopically expressing *PcDWF1* were plated on MS medium at pH 5.8 that was supplemented with 1% (w/v) sucrose and 0.7% (w/v) agar. After 15 days of growth, the seedlings were transplanted into soil. The temperature of the growth chamber was controlled at 23 ± 2 °C with a 16/8 h light/dark cycle, and the light intensity was 100 μmol·m^− 2^·s^− 1^.

### The exogenous BL treatment of the pear

For the BL treatment to the *Pyrus ussuriensis* plants, 0 mg/L, 0.1 mg/L, 0.5 mg/L, 1.0 mg/L, and 2.0 mg/L were added to the normal subculture medium, respectively. After 30 days of growth, the diameter and height of the stem were measured by a Vernier caliper. For the 0.5 mg/L BL treatment to the *Pyrus ussuriensis* plants. 0.5 mg/L BL was added to the normal subculture medium and the rooting medium, respectively. After 30 days of growth, a total of 10 BL-treated plants and 10 control plants under normal subculture medium were used to measure the diameter and height of the stem. For the rooting medium, along with the measurement of the diameter and the height of the stem, the number of roots was also measured. Each experiment was independently repeated three times.

To examine the effects of BL on the dwarf-type pears, a total of ten three-month-old dwarf-type pears and five standard-type pears were used. The five dwarf-type pears were sprayed with exogenous BL (0.5 mg/L) every 2 days. One month later, the plant height and internode length of the BL-treated dwarf-type pears, the control dwarf-type and standard-type pears were measured. All the materials were collected for further research. Each experiment was independently repeated three times.

### Phylogenetic analysis of the BR biosynthetic genes in pear and *Arabidopsis*

The rate-limiting BR biosynthetic genes in *Arabidopsis* were *AtDWF1*, *AtCPD*, *AtDWF4*, *AtBR6OX1*, and *AtBR6OX2*. The homologous genes in pear were BLASTed in the NCBI database. The phylogenetic tree was constructed with the neighbor-joining method and a bootstrap test with 1000 iterations based on the amino acid sequences of PCP020961.1 (PcDWF1), PCP044170.1, PCP005240.1, PCP016276.1 (PcDWF4–1), PCP021841.1 (PcDWF4–2), PCP011658.1 (PcCPD-1), PCP013214.1 (PcCPD-2), PCP026597.1 (PcBR6OX1–1), PCP012201.1 (PcBR6OX1–2), AtDWF1 (AT3G19820), AtCPD (AT5G05690), AtDWF4 (AT3G50660), AtBR6OX1 (AT5G38970), and AtBR6OX2 (AT3G30180) using MEGA5.2 software.

### Quantitative RT-PCR assay

For qPCR analysis of BR biosynthetic gene expression in dwarf-type (DW) and standard-type (WT) pears, *PcDWF1* expression under exogenous BL treatment, and BR biosynthetic gene expression in transgenic lines and wild type pear plants, total RNA was isolated from the pears using the RNAprep Pure Plant Kit (Tiangen, Beijing, China). The cDNA was synthesized using a PrimeScript™ RT reagent kit (Takara, DaLian, China). The LightCycler® 480 SYBR Green Master (Roche, Mannheim, Germany) was used for the qRT-PCR reactions on a LightCycler® 480 II system (Roche, Rotkreuz, Switzerland). *PcActin* was used as an internal control. The primer sequences for qPCR were designed based on the coding sequence of the BR biosynthetic genes, and they are shown in Table S1. Each experiment was independently repeated three times.

To conduct qPCR analysis of *PcDWF1* expression in transgenic tobacco lines and wild type tobacco, RNA extraction was performed using Trizol (TransGen Biotech, Beijing, China). The reverse transcription and the expression analysis were performed as described above. *NtActin* was used as an internal control. Each experiment was independently repeated three times.

### Determination of PcDWF1 subcellular localization

The CDS (coding domain sequence) and the promoter of *PcDWF1* were cloned from the leaves of the standard-type pear. The coding sequence without the stop codon of *PcDWF1* and the promoter of *PcDWF1* were inserted into the *pMDC83* vector to generate a *Pro PcDWF1*::*PcDWF1-GFP* construct. The primers are presented in Table S1. The *Pro PcDWF1::PcDWF1-GFP* plasmid was introduced into *Agrobacterium tumefaciens* strain GV3101. The leaves of one-month-old *Nicotiana benthamiana* plants were infiltrated with GV3101 harboring *Pro PcDWF1::PcDWF1-GFP*. FM4–64 (Invitrogen), as the plasma membrane marker, was dissolved in water and the tobaccos were labeled with 10 μM FM4–64 for 5 min, washed in MS liquid medium and observed immediately. The subcellular localization of PcDWF1-GFP was observed by confocal microscopy (× 40) (FV10-ASW, Olympus, Japan) 3 days after transient transformation.

### Generating transgenic tobacco and pear plants overexpressing *PcDWF1*

The *PcDWF1* open reading frame (ORF) was amplified using the primers presented in Table S1, and inserted into the pBI121 vector driven by the CaMV 35S promoter. To generate transgenic tobacco plants ectopically expressing *PcDWF1*, the *35S::PcDWF1* plasmid was introduced into *Agrobacterium tumefaciens* strain GV3101. One-month-old tobacco was selected for the transformation, which was performed as described by Wang et al. (2017) [68]. For the transformation of the pear, the *35S::PcDWF1* plasmid was introduced into *Agrobacterium tumefaciens* strain EHA105. One-month-old *Pyrus ussuriensis* plants were used for the transformation, which was performed as described by Yang et al. (2017) [69]. Genomic DNA was extracted from the leaves of the transgenic plants for PCR analysis. The levels of *PcDWF1* expression in all the transgenic tobacco and pear lines and control plants were determined by qPCR analyses. All primers are listed in Table S1.

### The light/dark treatment of the transgenic and control tobacco seedlings

To determine the effect of light on the growth of the transgenic and control tobacco plants, four transgenic lines (lines 3, 5, 6, and 8) with a high level of *PcDWF1* expression were selected for this experiment. The seeds of the transgenic tobacco overexpressing *PcDWF1* and the control tobacco plants were sown on MS medium. For the continuous light treatment, twenty wild type tobacco seeds and ten transgenic tobacco seeds from the *PcDWF1* overexpression lines 3, 5, 6, and 8 were placed under continuous light treatment for 10 days at a light intensity of 80 μmol·m^− 2^·s^− 1^. For the continuous dark treatment, the same amount of the seeds was grown under the dark treatment for 10 days. After 10 days of treatment, the length of the hypocotyls and the roots were measured, and the seedlings were photographed. Each experiment was independently repeated three times.

### The physiological parameters of the transgenic tobacco plants overexpressing *PcDWF1*

To determine the effect of *PcDWF1* overexpression on the growth of the tobacco plants, three transgenic lines (lines 3, 6, 8) with the highest expression level were selected. The transgenic tobacco plants and the control plants were transplanted into the soil after being grown on MS medium for 15 days. Fifty days later, the height of the stems was measured. After 90 days of growth in soil, the diameter and height of the stems were also measured. In addition, the weight and length of the roots were also measured. Each experiment was independently repeated three times.

### The physiological parameters of the transgenic pear plants overexpressing *PcDWF1*

To observe the growth of the plants, the transgenic pear lines overexpressing *PcDWF1* and the control plants were cultured on normal MS medium and rooting medium. For the growth of the pear in normal MS medium, 10 transgenic plants and 10 control plants were used for the evaluation, and the height of the plants was measured every 10 days during 50 days of culture. For the growth of the pear in rooting medium, the plant height and the root number were measured after 50 days of growth. Each experiment was independently repeated three times.

### Determination of BL content

To detect the endogenous BL concentrations of the transgenic lines and wild type tobacco and pear plants, three replicates of 2.0 g leaves, roots and stems were harvested, immediately frozen in liquid nitrogen, and then stored at − 80 °C until use. The extraction, purification, and determination of the endogenous levels of BL were performed by UPLC-MS technique as described by Zheng et al. (2018) [24]. Each experiment was independently repeated three times.

### Anatomical structure analysis of the transgenic and control tobacco plants

For the anatomical structure analysis of the tobacco plants, a razor blade was used to obtain sections of stem tissues from 90-day-old wild type and line 3 transgenic tobaccos, and the stem slices were observed with the aid of EVOS (Thermo Fisher, America). For the anatomical structure analysis of the pear plants, the stems of fifty-day-old transgenic pear and control plants were used for paraffin section. The sample preparation and observations of the cross-section and the longitudinal section of the stem were performed by light microscopy as described by Chen et al. (2015) [70]. Each experiment was independently repeated three times.

### Statistical analysis

The data were subjected to ANOVA followed by Fisher’s LSD or Student’s *t-*test. Statistically significant differences were indicated by *P* < 0.05. The statistical computations were conducted using SPSS software (IBM, Armonk, NY, USA).

## Supplementary information

**Additional file 1: Figure S1.** The effect of different concentrations of BL on the growth of *Pyrus ussuriensis* plants. **Figure S2.** Simplified brassinosteroid biosynthetic pathway with key rate-limiting enzymes (DWF1, CPD, DWF4, and BR6OX) involved in the reactions. **Figure S3.** Relative expression of BR biosynthetic rate-limiting genes in leaves, stems, roots of the dwarf-type and standard-type pears. **Figure S4.** The CDS sequence alignment of *PcDWF1* and the expression of *PcDWF1* under exogenous BL treatment. **Figure S5.** Relative expression of *PcDWF1* and the content of BL in transgenic lines and wild type tobacco plants under continuous light/dark treatment. **Figure S6.** Relative expression of *PcCPD-1*, *PcCPD-2*, *PcDWF4–1*, *PcDWF4–2*, *PcBR6OX1–1*, and *PcBR6OX1–2* in transgenic pear lines overexpressing *PcDWF1* and wild type pear plants.

**Additional file 2: Table S1.** The primers used for cloning, vector construction and qRT-PCR.

**Additional file 3.**

## Data Availability

All GenBank accession numbers from NCBI are AtDWF1 (Arabidopsis, NP_188616.1), AtCPD (Arabidopsis, NP_196188.1), AtDWF4 (Arabidopsis, NP_190635.1), AtBR6OX1 (Arabidopsis, NP_851105.1), AtBR6OX2 (Arabidopsis, NP_566852.1), respectively. The phylogenetic data was deposited into TreeBASE database, with the submission accession number (25767) and the URL (https://www.treebase.org/treebase-web/search/studySearch.html). The raw data are included in the supplementary information files. All data generated or analysed during this study are included in this published article [and its supplementary information files].

## Abbreviations

6-BA: 6-benzylaminopurine
BL: Brassinolide
BRs: Brassinosteroids
CDS: Coding domain sequence
CR: Campesterol
GFP: Green fluorescent protein
IBA: Indole-3-butyric acid
MS: Murashige and Skoog
ORF: Open reading frames
