# Supplementary material for: PcDWF1, a pear brassinosteroid biosynthetic gene homologous to AtDWARF1, affected the vegetative and reproductive growth of plants
Source: BMC Plant Biol. 2020 Mar 6;20:109. doi: 10.1186/s12870-020-2323-8 (PMC7060609; doi:10.1186/s12870-020-2323-8)
Supplement: Supplementary file 1 — Additional file 1: Figure S1. The effect of different concentrations of BL on the growth of Pyrus ussuriensis plants. Figure S2. Simplified brassinosteroid biosynthetic pathway with key rate-limiting enzymes (DWF1, CPD, DWF4, and BR6OX) involved in the reactions. Figure S3. Relative expression of BR biosynthetic rate-limiting genes in leaves, stems, roots of the dwarf-type and standard-type pears. Figure S4. The CDS sequence alignment of PcDWF1 and the expression of PcDWF1 under exogenous BL treatment. Figure S5. Relative expression of PcDWF1 and the content of BL in transgenic lines and wild type tobacco plants under continuous light/dark treatment. Figure S6. Relative expression of PcCPD-1, PcCPD-2, PcDWF4–1, PcDWF4–2, PcBR6OX1–1, and PcBR6OX1–2 in transgenic pear lines overexpressing PcDWF1 and wild type pear plants. [file 12870_2020_2323_MOESM1_ESM.pdf]

Additional files

Additional file 1

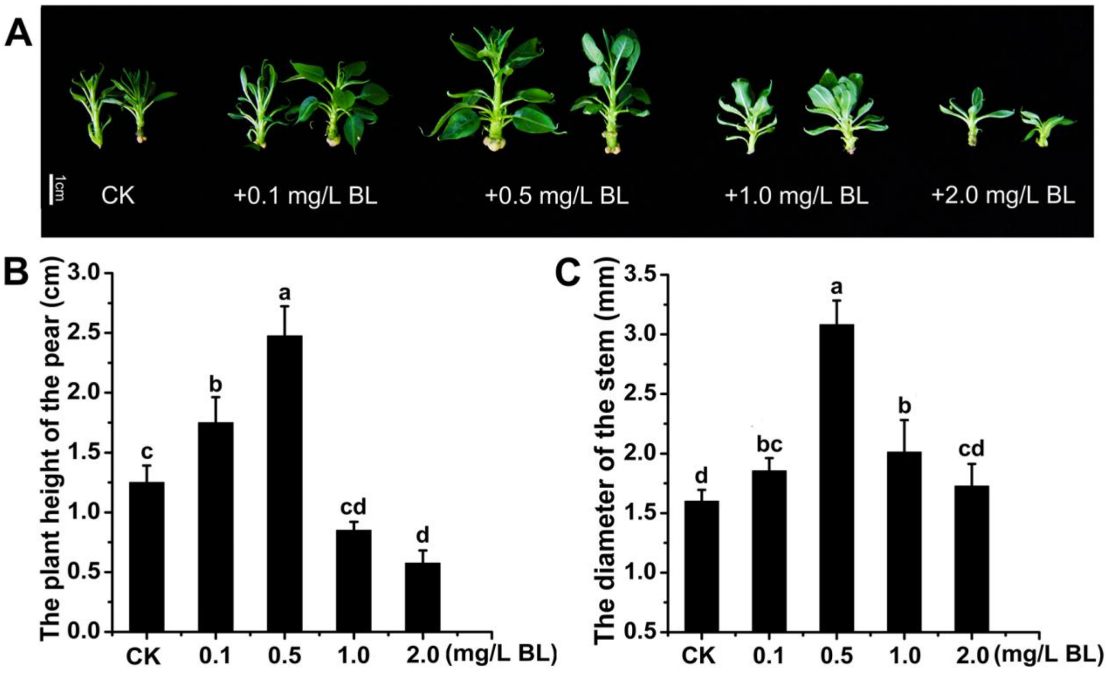

Figure S1 The effect of different concentrations of BL on the growth of *Pyrus ussuriensis* plants. (A) The phenotypes of *Pyrus ussuriensis* with 0 mg/L, 0.1 mg/L, 0.5 mg/L, 1.0 mg/L, and 2.0 mg/L BL treatment on normal medium for 30 days, respectively. The plant height (B) and the diameter of the stem (C) of *Pyrus ussuriensis* with different concentrations of BL treatment. Data are the means ± SD of triplicate experiments. Different lowercase letters indicate significant differences according to Fisher's LSD ($P < 0.05$).

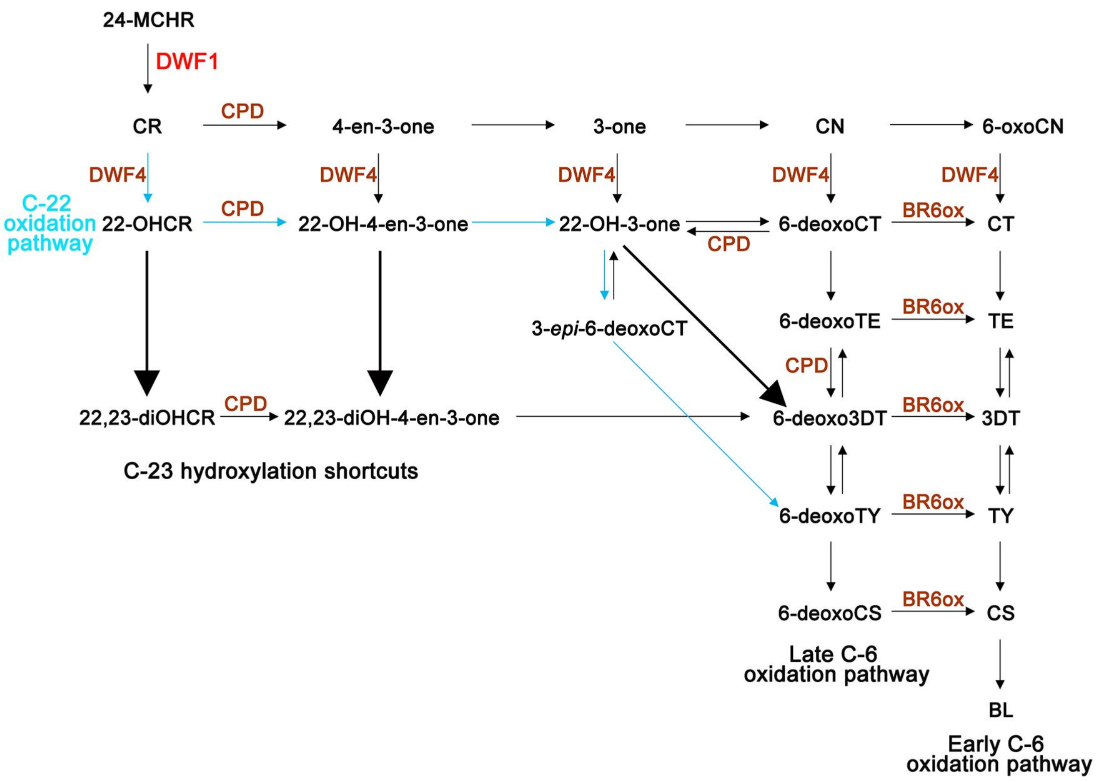

Figure S2 Simplified brassinosteroid biosynthetic pathway with key rate-limiting enzymes (DWF1, CPD, DWF4, and BR6OX) involved in the reactions.

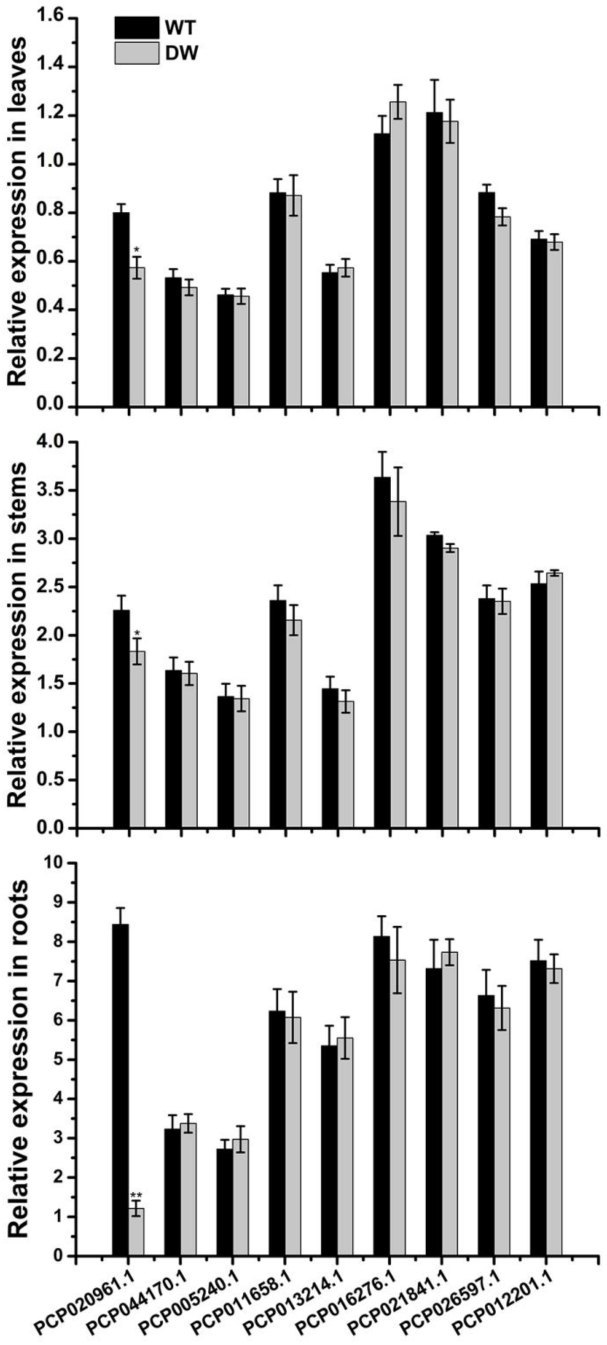

Figure S3 Relative expression of BR biosynthetic rate-limiting genes (*PCP020961.1*, *PCP044170.1*, *PCP005240.1*, *PCP016276.1*, *PCP021841.1*, *PCP011658.1*, *PCP013214.1*, *PCP026597.1*, and *PCP012201.1*) in leaves, stems, roots of the dwarf-type and standard-type pears. Data are the means ± SD of triplicate experiments. Asterisks (*) indicate significant differences from the control (Student's *t*-test, *$P < 0.05$, **$P < 0.01$).

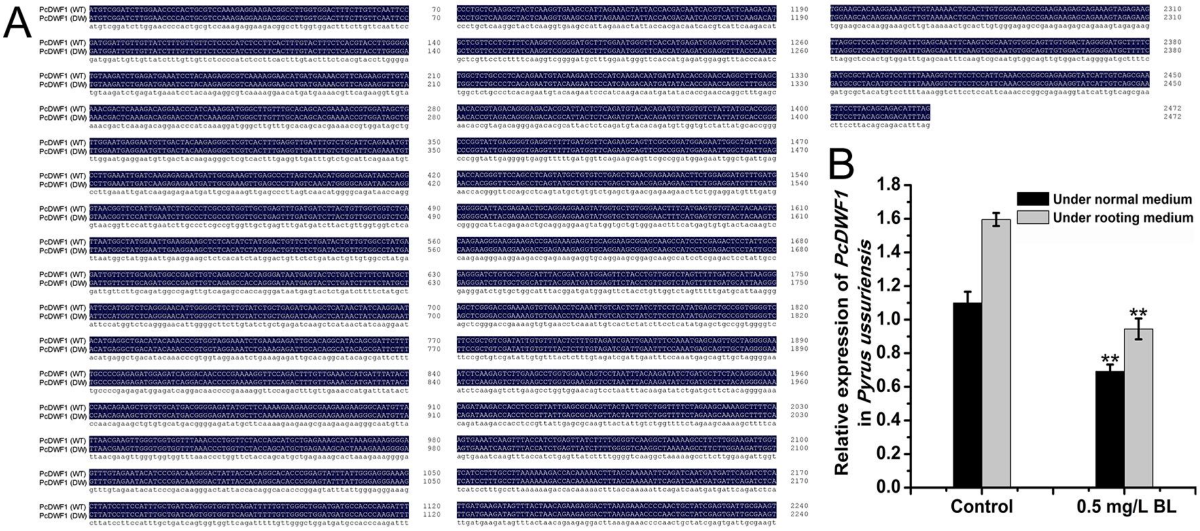

Figure S4 The CDS sequence alignment of *PcDWF1* and the expression of *PcDWF1* under exogenous BL treatment. (A) The CDS sequence alignment of the *PcDWF1* between the dwarf-type and standard-type pears. (B) Relative expression of *PcDWF1* in *Pyrus ussuriensis* plants with/without exogenous 0.5 mg/L BL treatment. Data are the means ± SD of triplicate experiments. Asterisks (*) indicate significant differences from the control (Student's *t*-test, $^{**}P < 0.01$).

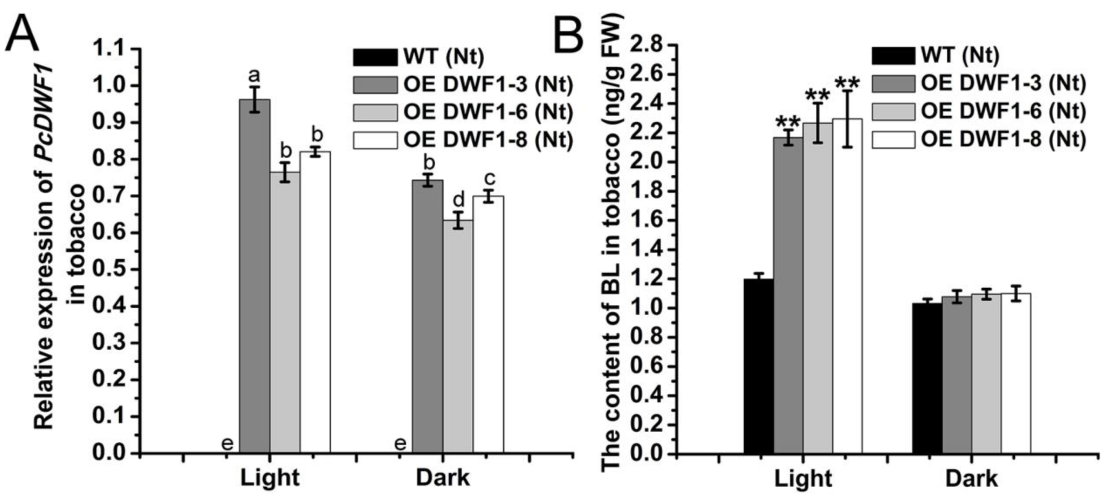

Figure S5 Relative expression of *PcDWF1* (A) and the content of BL (B) in transgenic lines and wild type tobacco plants under continuous light/dark treatment. Data are the means ± SD of triplicate experiments. Different lowercase letters indicate significant differences according to Fisher's LSD ($P < 0.05$). Asterisks (*) indicate significant differences from the control (Student's *t*-test, $^{**}P < 0.01$).

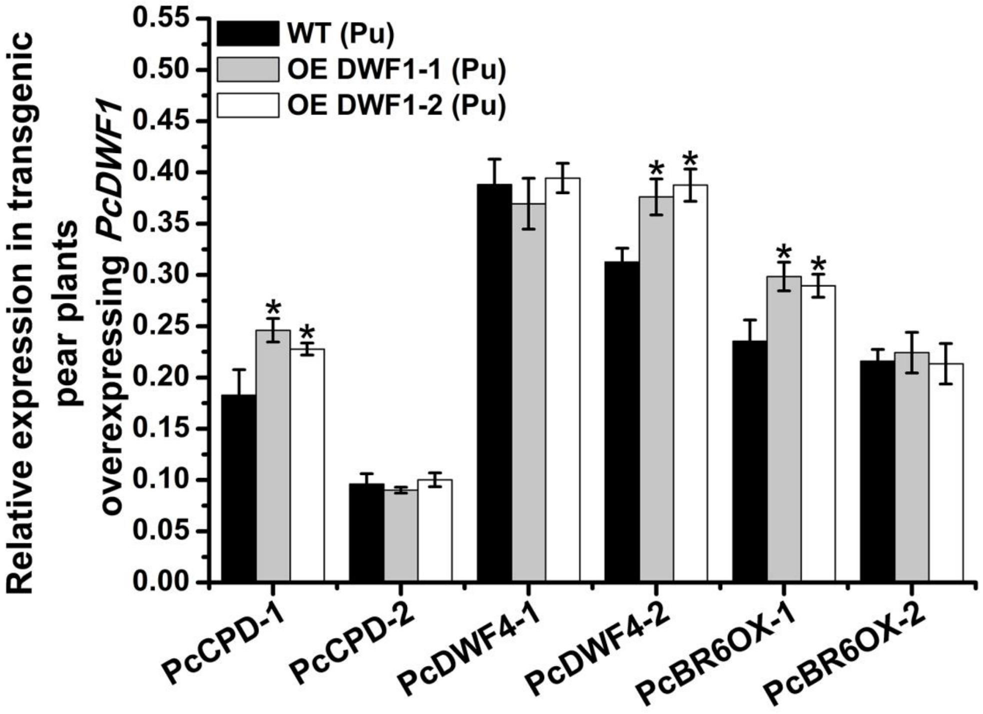

Figure S6 Relative expression of *PcCPD-1*, *PcCPD-2*, *PcDWF4-1*, *PcDWF4-2*, *PcBR6OX1-1*, and *PcBR6OX1-2* in transgenic pear lines overexpressing *PcDWF1* and wild type pear plants. Data are the means ± SD of triplicate experiments. Asterisks (*) indicate significant differences from the control (Student's *t*-test, *$P < 0.05$).

Additional file 2

Table S1 The primers used for cloning, vector construction and qRT-PCR

Primer name	Forward primer	Reverse primer	Vector
Cloning the CDS of *PcDWF1*	ATGTCGGATCTTGGAACC CCACTGC	CTAAATGTCTGCTGTAA GGAAGT	
Cloning the promoter of *PcDWF1*	ACACTCTTATCCTCTTTTA TCTAT	CAAGATCCGACATCTTG AAAGCA	
PcDWF1-GFP	TTAATTAAATGTCGGATC TTGGAACC	GGCGCGCCAAATGTCTG CTGTAAGG	pMDC83
Pro PcDWF1-pMDC83	AAGCTTACACTCTTATCC TCTTTTA	ACTAGTCTTGAAAGCAG AACCCTGT	pMDC83
PcDWF1-pBI121	GGATCCATGTCGGATCTT GGAACCC	CCCGGGAAATGTCTGCT GTAAGG	pBI121
PcActin for qPCR	TGGTGTCATGGTTGGTAT GG	CAGGAGCAACACGAAG TTCA	
NtActin for qPCR	AGAGGCCCTCAGACAAA C	TAGGTCCAAAGGTCAC AA	
PcDWF1 for qPCR	CAACACCGTAGACAGGG AGACA	CCGTAAATGCCAGCACA GATC	
PCP005240.1 for qPCR	CGGAGTATTTATTGGGAG GGAAAG	ACCTTGAGTAGCCTTGA GCAGA	
PCP011658.1 for qPCR	TCTCCGTTCCCTTGCCTC TATT	TCCCTCCTTTGCCTCAC TATCA	
PCP012201.1 for qPCR	CCTAATGAATGAAGCCA AAGGG	GAATGTTGATAACTTGG TTGTCCC	
PCP013214.1 for qPCR	CTCTAGTTTCCACCACCT ACCGC	CCAGAGGCTTTGACGA CTTCTTT	
PCP016276.1 for qPCR	ATGGAGATGGCAGCAGA ATAAC	TGAGGACAAGGTGGTG GATAAA	
PCP021841.1 for qPCR	AAGGATGTTCGGTACAA AGGGTA	ATGGATTGAAGTGTTGA GGGTG	
PCP026597.1 for qPCR	CTGGGACAACCAAGTCA TCAAC	GGTCTCATAACCCGAAT ACAAAA	
PCP044170.1 for qPCR	TTTTCTATGCTATTCCGTG GTCT	CCTGTGGTAGTAGTCCC TTGTCG	
