# Supplementary material for: PcDWF1, a pear brassinosteroid biosynthetic gene homologous to AtDWARF1, affected the vegetative and reproductive growth of plants
Source: BMC Plant Biol. 2020 Mar 6;20:109. doi: 10.1186/s12870-020-2323-8 (PMC7060609; doi:10.1186/s12870-020-2323-8)
Supplement: Supplementary file 2 [file 12870_2020_2323_MOESM2_ESM.docx]

Additional file 2

Table S1 The primers used for cloning, vector construction and qRT-PCR

Primer name	Forward primer	Reverse primer	Vector
Cloning the CDS of *PcDWF1*	ATGTCGGATCTTGGAACCCCACTGC	CTAAATGTCTGCTGTAAGGAAGT	
Cloning the promoter of *PcDWF1*	ACACTCTTATCCTCTTTTATCTAT	CAAGATCCGACATCTTGAAAGCA	
PcDWF1-GFP	TTAATTAAATGTCGGATCTTGGAACC	GGCGCGCCAAATGTCTGCTGTAAGG	pMDC83
Pro PcDWF1-pMDC83	AAGCTTACACTCTTATCCTCTTTTA	ACTAGTCTTGAAAGCAGAACCCTGT	pMDC83
PcDWF1-pBI121	GGATCCATGTCGGATCTTGGAACCC	CCCGGGAAATGTCTGCTGTAAGG	pBI121
PcActin for qPCR	TGGTGTCATGGTTGGTATGG	CAGGAGCAACACGAAGTTCA	
NtActin for qPCR	AGAGGCCCTCAGACAAAC	TAGGTCCAAAGGTCACAA	
PcDWF1 for qPCR	CAACACCGTAGACAGGGAGACA	CCGTAAATGCCAGCACAGATC	
PCP005240.1 for qPCR	CGGAGTATTTATTGGGAGGGAAAG	ACCTTGAGTAGCCTTGAGCAGA	
PCP011658.1 for qPCR	TCTCCGTTCCCTTGCCTCTATT	TCCCTCCTTTGCCTCACTATCA	
PCP012201.1 for qPCR	CCTAATGAATGAAGCCAAAGGG	GAATGTTGATAACTTGGTTGTCCC	
PCP013214.1 for qPCR	CTCTAGTTTCCACCACCTACCGC	CCAGAGGCTTTGACGACTTCTTT	
PCP016276.1 for qPCR	ATGGAGATGGCAGCAGAATAAC	TGAGGACAAGGTGGTGGATAAA	
PCP021841.1 for qPCR	AAGGATGTTCGGTACAAAGGGTA	ATGGATTGAAGTGTTGAGGGTG	
PCP026597.1 for qPCR	CTGGGACAACCAAGTCATCAAC	GGTCTCATAACCCGAATACAAAA	
PCP044170.1 for qPCR	TTTTCTATGCTATTCCGTGGTCT	CCTGTGGTAGTAGTCCCTTGTCG	
